# Advances in temporizing treatment strategies and associated complications for posthemorrhagic hydrocephalus in preterm infants

**DOI:** 10.3389/fped.2026.1864101

**Published:** 2026-07-30

**Authors:** Mengzi Tang, Guanchu Chen, Xue Liu, Qianqian Liu, Bin Yi, Hongxia Gao

**Affiliations:** 1 The First Clinical Medical College of Gansu University of Chinese Medicine, Lanzhou, China; 2 Department of Neonatology, Gansu Provincial Maternity and Child-care Hospital (Gansu Provincial Central Hospital), Lanzhou, China

**Keywords:** hydrocephalus, infant, premature, intraventricular hemorrhage, posthemorrhagic hydrocephalus, postoperative complications

## Abstract

Posthemorrhagic hydrocephalus (PHH) is a severe complication of intraventricular hemorrhage in preterm infants. It can cause varying degrees of brain injury, neurological impairment, and even death. The pathogenesis of PHH is complex, and several temporizing treatment strategies are currently available. However, these approaches differ in their associated complications and long-term outcomes, and no consensus has been reached regarding the optimal temporizing intervention. This review summarizes the procedural characteristics, complications, and prognostic outcomes of current temporizing treatments for PHH. It also discusses the major challenges, ongoing controversies, and recent advances in PHH management, with the aim of informing clinical decision-making.

## Introduction

Posthemorrhagic hydrocephalus (PHH) is a serious complication of intraventricular hemorrhage in preterm infants and may result in progressive brain injury and long-term neurological sequelae. Although several temporizing surgical interventions are available, no clinical consensus has been established regarding their optimal selection and application.

## Background

Preterm infants are particularly susceptible to intraventricular hemorrhage (IVH) because of the immaturity of the cerebral vasculature, and its incidence increases with decreasing gestational age. Following IVH, posthemorrhagic hydrocephalus (PHH) may develop through several mechanisms, including mechanical obstruction by blood clots, the release of inflammatory mediators, and transforming growth factor-*β*-mediated fibrosis (1). Studies have shown that PHH develops in approximately 9% of preterm infants with IVH, with reported incidence rates of 1%, 4%, 25%, and 28% among infants with grade I, II, III, and IV IVH, respectively (2). If left untreated, PHH can cause progressive ventricular dilation, leading to white matter and deep gray matter injury, reduced cerebellar volume, and subsequent neurological sequelae (3).

Current management strategies for PHH include temporizing CSF diversion, permanent shunting, and medical therapy. Because early placement of a permanent ventriculoperitoneal shunt (VPS) is associated with numerous complications, increasing attention has been directed toward temporizing interventions. However, selecting an appropriate temporizing procedure requires careful consideration of both its efficacy and the risk of complications. This review summarizes current temporizing interventions, including serial lumbar puncture (LP), external ventricular drainage (EVD), ventriculosubgaleal shunt (VSGS), ventricular access device (VAD), neuroendoscopic lavage (NEL), and drainage, irrigation, and fibrinolytic therapy (DRIFT), as well as their associated complications. The review aims to provide a reference for the clinical management of PHH.

We systematically searched four electronic databases, namely PubMed/MEDLINE, Embase, Web of Science, CNKI and the Cochrane Library, from inception through May 2026. The search terms and their derivatives included “posthemorrhagic hydrocephalus”, “PHH”, “posthemorrhagic ventricular dilatation”, “preterm”, “premature infants”, “temporary”, “temporizing”, “CSF diversion”, “ventricular access device”, “VAD”, “Ommaya reservoir”, “VSGS”, “external ventricular drainage”, “EVD”, “neuroendoscopic lavage”, “NEL”, “drainage irrigation and fibrinolytic therapy”, “DRIFT”, and “lumbar puncture”.

Studies were included if they met the following criteria: (1) the study population comprised preterm or low-birth-weight infants; (2) participants underwent at least one temporizing CSF diversion procedure, including LP, EVD, VAD, VSGS, NEL, or DRIFT; (3). at least one of the following outcomes was reported: complications, subsequent VPS placement, spontaneous resolution of hydrocephalus, mortality, or neurodevelopmental outcomes; (4). the publication was a primary study, including a randomized controlled trial, prospective or retrospective cohort study, case-control study, or case series; a secondary study, including a systematic review, meta-analysis, or narrative review; a clinical guideline; an expert consensus statement; or a technical report.

The exclusion criteria were as follows: (1) study populations other than preterm infants, including term infants, children, and adults; (2) hydrocephalus unrelated to intracranial hemorrhage, such as congenital, postinfectious, or tumor-associated hydrocephalus; (3) treatment with pharmacotherapy alone without surgical intervention; (4) single-case reportsor studies including fewer than three patients, except formally published technical reports with substantial demonstrative value, as well as conference abstracts, editorials, and commentaries; and (5). publications without extractable outcome data or duplicate reports.

## Temporizing treatment strategies for PHH and their complications

### Serial LP

Serial LP reduces intracranial pressure (ICP) and relieves brain parenchymal compression by removing hemorrhagic cerebrospinal fluid (CSF) and decreasing protein deposition within CSF circulation and reabsorption pathways. It may serve as a bridging therapy for PHH while preparations are made for subsequent neurosurgical procedures, such as VAD, VSGS, or EVD placement (4). Studies have shown that short-term serial LP may alleviate hydrocephalus-related symptoms in some infants with mild PHH, limited hemorrhage, and no evidence of obstruction (5). However, other studies have demonstrated that LP may provide insufficient CSF drainage and may therefore fail to control progressive ventricular dilation. Serial LP has also failed to reduce the need for subsequent VPS placement or improve long-term neurodevelopmental outcomes (6). Accordingly, LP is not recommended as the primary treatment for PHH. It should be used only as a short-term bridging measure and is generally limited to no more than three procedures (7). Moreover, because ventricular enlargement may progress rapidly in some infants with IVH, serial LP is no longer the preferred temporizing treatment for PHH in current clinical practice.

### EVD

EVD is a conventional temporizing procedure for PHH. It continuously drains CSF to reduce ICP, decrease ventricular dilation, and relieve compression of the brain parenchyma. In infants with concomitant ventriculitis or encephalitis, medications may also be administered intraventricularly through the EVD catheter (8). In a study examining the timing of EVD and subsequent neurodevelopmental outcomes in preterm infants, Bassan et al. (9) found that children who underwent early intervention at ≤25 days of age had better adaptive, personal-social, communication, and cognitive outcomes at 6 years of age than those who underwent later EVD placement. Percutaneous transfontanellar EVD is a modified EVD technique in which the drainage catheter is inserted through an open fontanelle. This approach is particularly suitable for extremely low-birth-weight infants (ELBWIs) (10). Unlike conventional EVD, this technique creates a subcutaneous tunnel using a venous catheter to connect the intracranial drainage port adjacent to the fontanelle with a scalp exit site in the temporal region. Subcutaneous tunneling of part of the catheter may reduce the risk of retrograde infection during drainage (10, 11).

EVD is associated with several complications, of which meningitis and ventriculitis are the most common infections. Diagnostic criteria vary among countries. Some definitions rely solely on positive CSF cultures, whereas others incorporate microbiological findings, clinical manifestations, and CSF parameters. Consequently, reported infection rates range from <5% to 20% (12). Risk factors for EVD-associated infection include contamination by skin flora, contamination of the drainage system, repeated EVD placement, prolonged catheterization, an inadequately long subcutaneous tunnel, and failure to administer perioperative prophylactic antibiotics (13). An indwelling duration of more than 5 days has been identified as a significant risk factor for infection, possibly because of biofilm formation on the catheter or hematogenous seeding from a systemic infection (14).

Other EVD-related complications include catheter obstruction, disconnection, and excessive CSF drainage, which may result in subdural effusion or hyponatremia (15). Hemorrhage is another important complication. One study reported hemorrhage rates of 21.6% during EVD insertion and 22.5% during catheter removal. Thrombocytopenia and repeated EVD placement further increased this risk (16).

More recently, Rahul Lakshmanan et al. (17) retrospectively evaluated 51 infants younger than 12 months who underwent CSF shunt placement. Widespread hemorrhages in infants postshunting (WHIPS) occurred in 16% of the cohort, exceeding the previously reported incidence of 2.9%–9.1% (18). The occurrence of WHIPS was associated with underlying conditions such as PHH and postinfectious hydrocephalus. Hemorrhagic lesions predominantly involved the corticocortical region, periventricular white matter, and deep white matter. Injury to these regions may substantially affect neurological outcomes. WHIPS should therefore be recognized as a distinct and clinically relevant complication in infants with PHH undergoing CSF diversion.

EVD can rapidly reduce ICP in urgent situations. However, because of its relatively high risk of infection and the intensive postoperative care required, it is not considered a first-line temporizing intervention and is generally reserved for selected clinical circumstances (19). Antibiotic-impregnated EVD catheters may reduce the risk of infection when this procedure is necessary (20). In addition, ultrasound-guided visualization of the lateral ventricular anatomy facilitates accurate catheter placement while avoiding blood vessels and functionally important brain regions. This technique may improve the safety of EVD as a temporizing treatment for PHH (21).

### VAD

A VAD consists of a drainage catheter inserted into the lateral ventricle and connected to a subcutaneous reservoir, (e.g., Ommaya reservoir or Rickham reservoir), which is secured beneath the galea aponeurotica to create a closed drainage system. CSF is withdrawn through the reservoir to reduce ICP, typically at a rate of 1 mL/min, with the volume removed during a single session not exceeding 10 mL/kg. Cranial ultrasonography should be performed at least twice weekly to monitor changes in ventricular size (22). Compared with LP or EVD, VAD can be implanted at the bedside in the neonatal intensive care unit (NICU), avoids repeated puncture-related trauma, and does not require long-term management of an external drainage system. In addition, VAD allows flexible adjustment of the CSF withdrawal volume according to changes in ventricular pressure (23).

Commonly used VADs include the Ommaya reservoir and the Rickham reservoir. The Ommaya reservoir is a standard universal subcutaneous reservoir device with a relatively large capsule size. It is primarily designed for use in adults and older children, and is mainly used for intrathecal chemotherapy for central nervous system tumors, and is less commonly used in neonates (24). By contrast, the Rickham reservoir is a neonatal-specific miniaturized VAD (25, 26). It features a steel puncture-resistant base and a thin, lightweight capsule, designed specifically for preterm infants <32 weeks of gestation with very low or extremely low birth weight. Its design accommodates the thin, fragile scalp and immature, small calvarium of preterm infants, and it is primarily used for the management of PHH in preterm infants (27).

Clinical studies have reported that 76.1% of preterm infants with PHH who received VAD intervention ultimately still required VPS placement, with CSF lactate level being the principal risk factor (28). In addition, VAD is associated with various postoperative complications. The reported incidence of CSF leakage is 22%, primarily attributed to inadequate reservoir fixation, poor wound healing, and weakening of the tissue at the puncture site after repeated needle access (13). Secondary intracranial hemorrhage has also been reported in clinical cases, possibly resulting from ventricular collapse caused by excessively rapid drainage or removal of large volumes of CSF, which stretches cerebral vessels and leads to parenchymal or subependymal hemorrhage (29). Percutaneous reservoir puncture for CSF withdrawal is associated with an infection risk of approximately 5.5%–8% (30). Studies have shown that standardized puncture protocols and multidisciplinary clinical pathways can reduce VAD-related infection rates from 4% to 0%, improve treatment adherence among infants with PHH, and ultimately improve clinical outcomes (23, 31, 32).

### VSGS

A VSGS diverts CSF into the subgaleal space, where it is gradually reabsorbed. This approach temporarily reduces intraventricular pressure and may limit pressure-related brain injury. By maintaining a closed system and isolating intracranial tissues from the external environment, VSGS reduces exposure to pathogens and may lower the risk of infectious complications. It also prevents the fluid and electrolyte losses associated with external drainage, thereby helping maintain fluid and electrolyte homeostasis in preterm infants (22). Cristina Bleil et al. (33) observed that the conventional technique, in which the subgaleal reservoir is positioned directly beneath the surgical incision, is associated with an increased risk of CSF leakage and infection. They modified the procedure by creating the subgaleal reservoir at a site distant from the incision, such as the occipital region, which substantially reduced the infection rate. A recent study by Syed I. Khalid et al. (34) demonstrated that preterm infants treated with VSGS were significantly less likely to require VPS placement within 6 months after surgery than those treated with VAD (*OR*, 0.22; 95% *CI*, 0.12–0.41; *P* < 0.001). This finding is consistent with the retrospective study by Kaushik Sil et al. (15) A meta-analysis by Daniel M. Fountain et al. (35) also found that VSGS reduced the need for subsequent VPS placement in pediatric patients.

Despite being a closed system, VSGS has several limitations. The capacity of the subgaleal space to accommodate and absorb CSF is limited. When CSF accumulation exceeds its absorptive capacity, the reservoir may enlarge and become tense, necessitating aspiration of fluid from the subgaleal cavity. The reservoir remains functional for an average of approximately 37 days. If the reservoir contracts or the device remains in place for more than 3 months, timely surgical revision may be required to reconstruct the subgaleal space (13).

The reported infection rate after VSGS placement ranges from 13.5% to 30.8% (33), particularly when the reservoir is created immediately adjacent to the incision. This configuration increases the likelihood of CSF leakage through the wound and facilitates invasion by skin flora, including Staphylococcus aureus (20). In addition, skull growth may cause catheter migration or dislodgement. The catheter may subsequently enter the brain parenchyma and cause tissue injury (36).

However, comparative studies of VSGS and VAD as temporizing treatments have found no significant differences in infection, obstruction, device dependence, or long-term neurodevelopmental outcomes (37). The choice of procedure should therefore be individualized according to the infant's clinical condition, the anticipated risk of surgical complications, and the surgeon's experience. Long-term neurodevelopmental outcomes and the likelihood of subsequent permanent shunt placement should also be considered.

### NEL

NEL uses a neuroendoscope and peel-away sheath to access the lateral ventricle, allowing direct evaluation of CSF characteristics and intraventricular adhesions. Lactated Ringer's solution is used for irrigation, and forceps may be used to remove blood clots, inflammatory mediators, and proteinaceous debris adherent to the choroid plexus or ventricular wall. Septostomy may then be performed to permit lavage of the contralateral ventricle. Irrigation flow must be carefully controlled to avoid traumatic injury to the brain parenchyma, minimize cerebral damage, and reduce the risk of slit ventricle formation (38).

NEL is generally considered for infants with PHH who do not respond to conventional treatment or require repeated interventions. The principal indications include the following (39): (1) clinical manifestations of increased ICP, such as a tense fontanelle, vomiting, bradycardia, or respiratory distress; (2) an excessive increase in head circumference, defined as a weekly increase ≥14 mm or a mean daily increase ≥2 mm; and (3) ultrasonographic evidence of ventricular enlargement, including a ventricular index (VI) >97th percentile +4 mm, anterior horn width (AHW) >97th percentile +1 mm, thalamo-occipital distance (TOD) >97th percentile +1 mm, or third ventricular width >97th percentile +1 mm.

Potential complications of NEL include secondary infection, CSF leakage, and shunt dysfunction. A small proportion of patients may require repeat lavage because of progressive ventricular enlargement, residual intraventricular blood clots, or newly developed hematomas (39, 40). Current evidence does not indicate that NEL increases the long-term risk of neurological disorders such as epilepsy.

A 2023 meta-analysis compared ventricular lavage using NEL or DRIFT with other treatments, including VPS, EVD, LP, and VSGS. Ventricular lavage was associated with lower rates of subsequent VPS placement and infection than the comparator treatments (*OR*, 0.22 and 0.20; *95% CI*, 0.05–0.97 and 0.07–0.59; *P* = 0.05 and *P* < 0.05, respectively). However, no significant difference was observed in severe neurological outcomes (*OR*, 0.99; *95% CI*, 0.13–7.23; *P* = 0.99) (41). A meta-analysis by Kandula et al. (42) further showed that NEL was safer than DRIFT, particularly with respect to secondary hemorrhage (6.9% vs. 20.0%, *P* < 0.001). These findings support NEL as the preferred approach for intraventricular clot evacuation in preterm infants with severe IVH, particularly grade III or IV disease, and may help reduce the inappropriate use of higher-risk techniques such as DRIFT.

A Turkish multicenter study classified 74 infants with PHH into three groups according to treatment modality and timing: early NEL (*n* = 23), conventional temporizing CSF diversion (*n* = 29), and VSGS (*n* = 22). Compared with the other two approaches, NEL promoted earlier clearance of intraventricular blood and its degradation products, thereby reducing blood-induced inflammation. It was also associated with lower rates of subsequent VPS placement (60.8% vs. 93.1% vs. 77.2%, *P* = 0.019), CSF infection (4.3% vs. 27.5% vs. 36.6%, *P* = 0.029), and multiloculated hydrocephalus (8.6% vs. 34.4% vs. 40.9%, *P* = 0.037) (43).

NEL may be performed as a standalone procedure or combined with EVD for ICP monitoring, followed by a gradual transition to intermittent drainage or VSGS placement. A retrospective study by Hall et al. (44) found that NEL alone was associated with a lower risk of wound infection than combined NEL and VSGS. Conversely, other studies have indicated that NEL combined with endoscopic third ventriculostomy may reduce the need for VPS placement compared with NEL alone (*RR*, 0.500; *95*% *CI*, 0.315–0.794; *P* = 0.0033) (38).

More recently, Mediratta et al. (45) initiated the ENLIVEN-UK study, a nationwide, multicenter, parallel-group, assessor-blinded, phase III randomized controlled trial. The trial is designed to evaluate the efficacy and safety of NEL combined with VAD or VSGS vs. VAD or VSGS alone for the treatment of posthemorrhagic ventricular dilatation (PHVD) in preterm infants with IVH. The primary outcome is the cognitive quotient at 2 years of corrected age. This trial is intended to address the lack of prospective comparative evidence supporting NEL in this population. If the intervention proves effective, NEL may be incorporated into standard treatment pathways, potentially improving neurodevelopmental outcomes and reducing the need for permanent VPS placement. At present, the decision to use NEL alone or in combination with another procedure should be based on the infant's clinical condition and the expertise and resources available at the treating center.

Recent studies have also examined long-term outcomes after NEL in children with PHH. Behrens et al. (46) conducted a retrospective cohort study of 45 children treated with NEL. Neurocognitive development at 2 years of age was assessed using the Bayley Scales of Infant and Toddler Development, Second Edition, and walking ability was evaluated using the Gross Motor Function Classification System. Among the children with available follow-up data, 28 (78%) were able to walk independently or with minimal assistance, and 8 (30%) achieved neurocognitive development approaching the normal range. These findings suggest that NEL may improve motor outcomes, although its effectiveness in preventing severe cognitive impairment remains limited.

As a minimally invasive technique, NEL may be further improved through appropriate selection and refinement of endoscopic equipment. The outer diameter of the endoscope, size of the working channel, and quality of visualization may influence surgical outcomes. Future endoscopic systems are likely to incorporate smaller outer diameters, higher-quality optics, balanced irrigation systems, and compatibility with multifunctional instruments (47). Nevertheless, the clinical application of NEL remains at an early stage. High-quality studies are needed to define its optimal indications, timing, and combination with other temporizing procedures and to clarify its role in the management of PHH in preterm infants.

### DRIFT

DRIFT is an novel therapeutic for PHH. Under ultrasonographic or computed tomography (CT) guidance, temporizing catheters are inserted through the frontal and occipital regions. The frontal catheter is used for the continuous infusion of artificial CSF and fibrinolytic agents, whereas the occipital catheter is used for drainage. The primary objective is to remove intraventricular blood, clots, and neurotoxic substances, including free iron and inflammatory mediators, thereby reducing secondary brain injury. During irrigation, the flow rate is adjusted according to the color of the drainage fluid, with an initial rate of 1–2 mL/h. ICP should be maintained below 7 mmHg (95.2 mmH_2_O) throughout treatment. Drainage is continued for 72 h or until the effluent becomes clear (48). During DRIFT, the VI and CSF characteristics should be closely monitored. Catheter removal may be considered when the following criteria are met: a negative light-transmission test of the drainage fluid for 24 consecutive hours, *a* ≥ 20% reduction in ventricular volume relative to pretreatment measurements on ultrasonography or CT, and maintenance of ICP within the normal range (49, 50).

Fibrinolytic agents used in DRIFT include urokinase and recombinant tissue plasminogen activator (rtPA). By activating plasminogen and promoting fibrin degradation, rtPA may facilitate efficient clearance of intraventricular clots. In the phase I DRIFT trial, infants treated with rtPA exhibited a 30% faster rate of intraventricular clot dissolution than those treated with lavage alone, together with a greater reduction in CSF ferritin levels. Neuroimaging also demonstrated a lower rate of progressive ventricular enlargement in the rtPA group relative to standard treatment (48).

However, in a phase II randomized trial, secondary IVH occurred in 35% of infants receiving rtPA, compared with 8% in the standard treatment group. This complication was attributed to systemic fibrinolytic activation, reduced fibrinogen levels, and further destabilization of the already fragile coagulation system in preterm infants (49). At 10-year follow-up, children in the rtPA group had a cognitive quotient 15.6 points higher than that of children receiving standard treatment, suggesting that DRIFT may have neuroprotective effects. However, this benefit does not outweigh the significant short-term bleeding risk (50).

Because of the substantial hemorrhagic risk associated with rtPA, recent DRIFT protocols have increasingly incorporated urokinase. Urokinase converts plasminogen to plasmin and promotes fibrin degradation while exerting a relatively limited effect on circulating fibrinogen. Park et al. (51) evaluated 43 infants with PHH and classified them into an early EVD group (*n* = 25), a late EVD group (*n* = 5), and a conservative-treatment group (*n* = 13). Of the infants in the early-intervention group, 21 received ventricular lavage consisting of EVD combined with intraventricular administration of 6,000 IU of urokinase every 3–6 h. Eighteen infants (86%) avoided subsequent VPS placement, and no severe complications, such as hemorrhage or infection, were reported. At a corrected age of 36 months, the 17 infants who received early intervention had better motor and cognitive-language outcomes than the 15 infants who underwent late intervention or conservative management (86% vs. 40%, *P* = 0.036). These findings suggest that urokinase may be more compatible with the coagulation characteristics of neonates. However, this conclusion derives from a single-center, small-sample cohort, with limited evidentiary quality; its efficacy and safety require further validation.

DRIFT is generally considered for preterm infants born at ≤32 weeks of gestation who develop PHVD, grade III–IV IVH, or a VI ≥97th percentile+4 mm (49). Nevertheless, DRIFT carries multiple notable complications. When lavage catheters remain in place for more than 72 h, the incidence of ventriculitis may reach 8% (51). Improper irrigation or excessive flow rates may cause fluctuations in ICP, potentially resulting in acute cerebral edema or hypotension (50). Administration of rtPA and mechanical irritation from the catheter may induce secondary intracranial hemorrhage. In addition, blood clots or fibrin deposition may obstruct the catheter and necessitate repeated repositioning (48).

Strict aseptic technique is therefore required during DRIFT catheter placement and subsequent management. CSF white blood cell counts should be monitored regularly. If ventriculitis is suspected, CSF cultures should be obtained promptly, and antibiotic therapy should be initiated when indicated. If hemorrhage occurs, fibrinolytic therapy should be temporarily discontinued and replaced with saline irrigation while any underlying coagulation abnormalities are actively corrected (49).

DRIFT may, to some extent, reduce the short-term need for VPS placement and may also confer some long-term cognitive benefits. However, compared with NEL, LP, and other procedures, DRIFT is technically demanding, requires specialized equipment and expertise, and carries relatively high risks of intracranial hemorrhage and ventriculitis. It is therefore not recommended as a routine first-line intervention for PHH. At present, the efficacy and safety of DRIFT still lack validation from multicenter, large-sample randomized controlled trials. Standardized protocols and individualized drug selection strategies still require additional clinical data.

The principal advantages, complications, and subsequent VPS placement rates associated with these six temporizing procedures are summarized in Table 1. A comprehensive comparison of the major complication rates associated with these interventions is presented in Table 2.

**Table 1:** Key characteristics and clinical outcomes of temporizing surgical procedures for PHH in preterm infants.

Procedure	Key Advantages	Main Complications/Risks	Rate of subsequent VPS	Key Evidence/Outcome
Serial LP	Simple, transitional bridging therapy	Inadequate drainage, rapid ventricular enlargement, no improvement in VPS rate or neurodevelopment	Not reduced	Not recommended as primary treatment; ≤3 procedures only (7)
EVD	Rapid ICP reduction, allows intraventricularmedication	Infection (5%–20%), hemorrhage (21.6% placement/22.5% removal), WHIPS (16%), overdrainage, subdural effusion, hyponatremia	High	Early intervention (≤25 d) improves cognitive outcome; (9) PTTEVD reduces infection (10, 11)
VAD	Bedside implantation, no long-term external drainage, adjustable drainage volume	Infection (5.5%–8%), CSF leakage (22%), secondary hemorrhage (ventricular collapse)	76.1%	CSF lactate ≥2.8 mg/dL is independent predictor for VPS conversion (28)
VSGS	Closed system, low pathogen exposure, stable fluid/electrolyte balance	Reservoir enlargement (mean 37 d), infection (13.5–30.8%), catheter displacement	Lower than VAD (OR 0.22, 95% CI 0.12–0.41, *P* < 0.001)	Modified VSGS reduces infection (33); similar neurodevelopment to VAD (37)
NEL	Lower VPS/infection/multifocal hydrocephalus rates; safer than DRIFT	Secondary infection, CSF leakage, rare repeat lavage	60.8%	Early NEL improves outcomes (43); NEL + ETV reduces VPS (38); ENLIVEN-UK RCT ongoing (45)
DRIFT	Removes neurotoxic substances; cognitive benefit observed (limited evidence)	Secondary hemorrhage (35% vs. 8% in controls), ventriculitis (>72 h: 8%), catheter obstruction, ICP fluctuation	No reduction vs. standard care; specific subgroup: 86% avoided with early urokinase（limited evidence）	rtPA: cognitive benefit but significant hemorrhage; urokinase: limited evidence; not recommended for routine use

PHH, posthemorrhagic hydrocephalus; ICP, intracranial pressure; VPS, ventriculoperitoneal shunt; EVD, external ventricular drainage; WHIPS, widespread hemorrhages in infants postshunting; VAD, ventricular access device; VSGS, ventriculosubgaleal shunt; NEL, neuroendoscopic lavage; DRIFT, drainage, irrigation, and fibrinolytic therapy; rtPA, recombinant tissue plasminogen activator; ETV, endoscopic third ventriculostomy; RCT, randomized controlled trial.

**Table 2:** Comparison of major complication rates among temporizing procedures for PHH.

Complication	LP	EVD	VAD	VSGS	NEL	DRIFT (rtPA)
Infection (meningitis/ventriculitis)	Low	<5%–20%	5.5%–8%	13.5%–30.8%	4.3%	8% (ventriculitis, >72 h)
CSF leakage	Rare	Occasional	22%	Common	Occasional	Not typical
Secondary Hemorrhage	Very low	21.6% (placement), 22.5% (removal), WHIPS 16%	Ventricular collapse induced	Low	6.9%	35% (vs. 8% in controls)
Catheter obstruction/dysfunction	Not applicable	Yes	Reservoir puncture related	Reservoir enlargement, displacement	Rare	Yes (clot/fibrin deposition)
Other notable complications	Inadequate drainage, rapid ventricular enlargement	Overdrainage, subdural effusion, hyponatremia	Tissue weakening from repeated punctures	Reservoir contraction (>3 months revision)	Repeat lavage	ICP fluctuation, cerebral edema, hypotension

LP, lumbar puncture; EVD, external ventricular drainage; VAD, ventricular access device; VSGS, ventriculosubgaleal shunt; NEL, neuroendoscopic lavage; DRIFT, drainage, irrigation, and fibrinolytic therapy; CSF,cerebrospinal fluid, ICP, intracranial pressure.

## Clinical guidelines, expert consensuses statements, and assessment of evidence quality

### International guidelines and expert consensuses statements

In 2014, the American Association of Neurological Surgeons and Congress of Neurological Surgeons (AANS/CNS) published the clinical guideline entitled “Pediatric Hydrocephalus: Systematic Literature Review and Evidence-Based Guidelines. Part 2: Management of PHH in Premature Infants.” (52) Based on 68 studies identified through PubMed and the Cochrane Library, the guideline issued seven graded clinical recommendations. Level II evidence supported VAD, EVD, VSGS, and LP as potential temporizing interventions for PHH. VSGS was also associated with a reduced need for daily CSF aspiration. Three recommendations based on Level I evidence advised against serial LP, intraventricular fibrinolytic therapy, and combined acetazolamide and furosemide therapy. Two Level III recommendations concluded that the available evidence was insufficient to determine the optimal timing of permanent shunt placement or support the use of endoscopic third ventriculostomy in preterm infants with PHH.

In 2022, a UK-based Delphi consensus established standardized surgical and perioperative nursing protocols for NEL in preterm infants with PHVD (53). The consensus recommended NEL for infants born at <37 weeks of gestation who developed severe ventricular dilatation. However, the proposed protocol has not yet been prospectively validated with respect to long-term neurodevelopmental outcomes, and standardized indications and intraoperative management strategies remain to be established.

China published its first systematic national expert consensus on PHVD and PHH in 2025 (54). Following a comprehensive review of the domestic and international literature, the consensus applied the GRADE framework to formulate standardized recommendations for disease surveillance, stratified intervention, and long-term prognostic management. It strongly recommended routine serial cranial ultrasonography in preterm infants as the principal imaging modality for the diagnosis and longitudinal assessment of PHH and PHVD.

### Assessment of evidence quality

A total of 72 relevant publications were included in this review: 2 randomized controlled trials (RCTs), 5 prospective studies, approximately 32 retrospective cohort studies and case series, 6 systematic reviews or meta-analyses, 4 clinical guidelines or expert consensus statements, 17 narrative reviews or expert opinion articles, and 6 preclinical studies or technical reports. The guidelines and consensus statements included the 2014 AANS/CNS guideline, the UK Delphi consensus on NEL, and the 2025 Chinese national expert consensus. Overall, 39 primary observational studies were included, accounting for approximately 54% of all eligible publications.

The overall quality of the available evidence remains limited, primarily because of the small number of RCTs. Most studies enrolled relativelyfew patients, with the majority including fewer than 100 participants, and were conducted at single centers. Follow-up generally extended only to a corrected age of 18–24 months. Long-term data on cognitive, behavioral, and functional outcomes during school age and adolescence remain scarce.

Synthesis of the available literature and current guidelines reveals substantial evidence gaps regarding temporizing treatment strategies for PHH in preterm infants. Considerable heterogeneity in the definitions of complications, concurrent comorbidities, treatment protocols, and follow-up duration limits direct comparisons among interventions. Robust randomized evidence is lacking for all major temporizing procedures, including VAD, VSGS, EVD, and NEL.Furthermore, inconsistent diagnostic criteria for adverse events, such as infection, device obstruction, and recurrent hemorrhage, substantially reduce the reliability of pooled meta-analytic estimates. Standardized frameworks for defining, assessing, and consistently reporting PHH-related complications are therefore urgently needed in future studies.

## Current research status and future directions in PHH treatment

### Current treatment challenges and controversies

No consensus has been established regarding the optimal treatment strategy or surgical management of PHH in preterm infants. The principal clinical controversies and remaining research gaps in the temporizing management of PHH are summarized in Table 3. These challenges involve three main aspects. First, PHH has a complex pathogenesis involving multiple pathological processes after IVH, including mechanical obstruction and inflammatory responses. Consequently, an intervention targeting a single mechanism may be insufficient for comprehensive disease management. Second, the efficacy of different treatments varies according to the severity of IVH. For example, LP may benefit infants with mild IVH but has limited effectiveness in severe cases. Whether other temporizing measures reduce the need for permanent shunting remains controversial because of interpatient variability, differences in healthcare practices, and surgeon-specific preferences regarding operative techniques. Third, surgical outcomes for PHH are generally less favorable than those for hydrocephalus caused by congenital anomalies or neural tube defects (55).

**Table 3:** Current clinical controversies and research gaps in the temporizing treatment of PHH.

Domain	Key Controversy/Gap	Proposed Direction/Ongoing Work
Early vs. late intervention	Europe favors early intervention (>97th percentile); North America prefers symptom-driven	Individualized early strategy based on dynamic ultrasound parameters
NEL indication and protocol	No unified criteria; wide center variation	UK expert consensus proposed three-stage protocol; ENLIVEN-UK phase III RCT ongoing
DRIFT risk-benefit	rtPA: cognitive benefit but significant hemorrhage (35% vs. 8%); urokinase: possibly safer, insufficient evidence; risk-benefit unclear	Optimize fibrinolytic agent selection, add neuroprotective agents, improve catheter systems
Stem cell therapy	Preclinical/phase I trials safe	Need large-scale RCTs to confirm efficacy for PHVD/PHH
Standardized protocols	No consensus on procedure selection, infection diagnosis, or nursing	Urgent need for international guidelines and multidisciplinary clinical pathways
Long-term neurodevelopment	Few comparisons at ≥2 years corrected age	Ongoing prospective trials (e.g., ENLIVEN-UK) will provide high-level evidence

PHH, posthemorrhagic hydrocephalus; NEL, neuroendoscopic lavage; DRIFT, drainage, irrigation, and fibrinolytic therapy; rtPA, recombinant tissue plasminogen activator; PHVD, posthemorrhagic ventricular dilatation; RCT, randomized controlled trial.

### Research advances and major controversies

Emerging treatment directions: In recent years, the goals of PHH treatment have shifted from reducing mortality and ensuring safe shunt placement toward avoiding permanent shunting and improving long-term neurological function. Treatment strategies have similarly evolved from passive CSF drainage to active clearance of intraventricular blood products, with increasing emphasis on minimally invasive and safer surgical approaches. Techniques such as NEL and DRIFT can remove intraventricular hematomas and blood degradation products, reduce inflammatory mediator levels, and mitigate secondary brain injury at its source (56). Accumulating evidence has also associated NEL with smaller ventricular size and improved neurodevelopmental outcomes (57). A recent UK expert consensus emphasized the standardization of indications and procedural protocols for NEL in preterm infants with PHVD. The proposed protocol covers patient selection, a three-stage operative approach comprising preventricular, intraventricular, and postventricular phases, and postoperative care. According to the consensus, NEL may be considered in preterm infants born before 37 weeks of gestation whose ventricular measurements exceed the 97th percentile+4 mm. However, the consensus also acknowledged the lack of prospective trials evaluating the effects of NEL on long-term neurodevelopment. Considerable variation persists in patient selection, operative techniques, and postoperative management. Large prospective RCTs are therefore needed to strengthen the current evidence base (53).

Stem cell therapy and targeted modulation of inflammation have also shown potential neurorestorative effects. Studies by Ahn and colleagues demonstrated that intraventricular transplantation of human umbilical cord-derived mesenchymal stem cells (MSCs) attenuated hydrocephalus and brain injury in neonatal rats (58). MSCs with preserved brain-derived neurotrophic factor activity reduced neuronal death, alleviated brain injury, and slowed the progression of hydrocephalus. In the first phase I clinical trial conducted by this group, nine preterm infants received escalating doses of MSCs. The treatment was well tolerated, with no severe adverse events or deaths reported (59). Nevertheless, large RCTs are required to clarify the safety, efficacy, and risk–benefit profile of stem cell therapy for PHVD and PHH (60).

Disagreement regarding the timing of PHH intervention: The central debate over early vs. late intervention concerns whether treatment should be initiated prophylactically or only after clinical symptoms develop. European practice generally favors early intervention when the VI exceeds the 97th percentile, with prophylactic ventricular drainage performed to reduce the risk of neurological injury. In contrast, North American practice tends to delay intervention until signs of intracranial hypertension emerge, such as a rapid increase in head circumference of >2 cm/week, a bulging fontanelle, or altered consciousness, to avoid unnecessary surgery (61). Early intervention may relieve pressure on the surrounding brain tissue caused by progressive ventricular enlargement and hydrocephalus, attenuate inflammatory responses and neurotoxicity, and prevent adverse effects on long-term neurodevelopment. A meta-analysis by Lai et al. involving 2,533 infants with PHVD found that delayed temporizing intervention was associated with higher rates of VPS placement and an increased risk of moderate-to-severe neurodevelopmental impairment (62). The randomized controlled Early Versus Late Ventricular Intervention Study (ELVIS) demonstrated that (63), although a low-threshold intervention strategy did not reduce the eventual need for VPS placement, long-term follow-up showed that early intervention reduced the composite risk of death or severe neurodevelopmental impairment at 2 years of age by 76%. These neuroprotective effects may result from mitigating mechanical and inflammatory injury to the developing white matter during the period of maximal ventricular dilatation.

Ultrasonography-guided approaches have been proposed to facilitate early intervention and outcome prediction. Serial monitoring of parameters such as the VI, AHW, and TOD may help determine the optimal timing of intervention (64). Studies have suggested that ultrasound-guided early intervention may reduce the VPS placement rate to 20%–25% (65), although its effectiveness requires confirmation in large-scale prospective trials (66). Future research should focus on identifying and validating specific ancillary examinations, dynamic monitoring strategies, and key indicators that can guide early intervention for PHH. The development of individualized early-intervention strategies based on these findings may reduce complication rates and improve long-term outcomes.

### Comparisons among temporizing therapeutic strategies

Temporizing CSF diversion is the cornerstone of PHH management, with the primary goals of controlling intracranial hypertension and allowing time for the spontaneous restoration of CSF circulation. High-quality evidence indicates that, when performed according to standardized clinical protocols, VAD and VSGS do not differ significantly in the avoidance of permanent VPS placement, overall infection rates, or long-term neurodevelopmental outcomes. Therefore, these procedures may be regarded as equivalent alternatives, with selection tailored to individual clinical circumstances (67, 68). By contrast, EVD permits precise regulation of drainage pressure and flow. However, its complex bedside management, higher risk of infection, and frequent need for device revision make it unsuitable for routine first-line use. EVD is therefore generally reserved for patients requiring strict ICP monitoring or salvage treatment (67, 69). NEL enables direct irrigation of the ventricular system under endoscopic visualization, facilitating the removal of intraventricular blood clots and inflammatory debris. Meta-analyses have shown that NEL reduces the risk of permanent shunt dependence by 78% and significantly decreases the incidence of infection and secondary multiloculated hydrocephalus. Nevertheless, because NEL remains an emerging technique, its safety and efficacy require further validation in multicenter RCTs (41).

### Clinical decision-making and future perspectives

The clinical management of neonatal PHH is shifting from experience-based practice toward evidence-based, individualized decision-making, which requires a comprehensive understanding of the underlying pathophysiological mechanisms. Traditional theories have primarily attributed PHH to mechanical obstruction caused by intraventricular blood clots. More recent studies, however, have revealed a substantially more complex pathological cascade. Intraventricular blood components trigger intense inflammatory responses that not only damage cerebral white matter but also promote excessive active CSF secretion through activation of the TLR4-SPAK/NKCC1 signaling pathway in choroid plexus epithelial cells. Together, these processes create a multifactorial pathological basis for impaired CSF circulation and progressive ventricular dilatation (66, 70, 71). These findings provide a strong biological rationale for early therapeutic strategies aimed at removing intraventricular hematomas and inflammatory mediators, including NEL.

**Figure 1:**
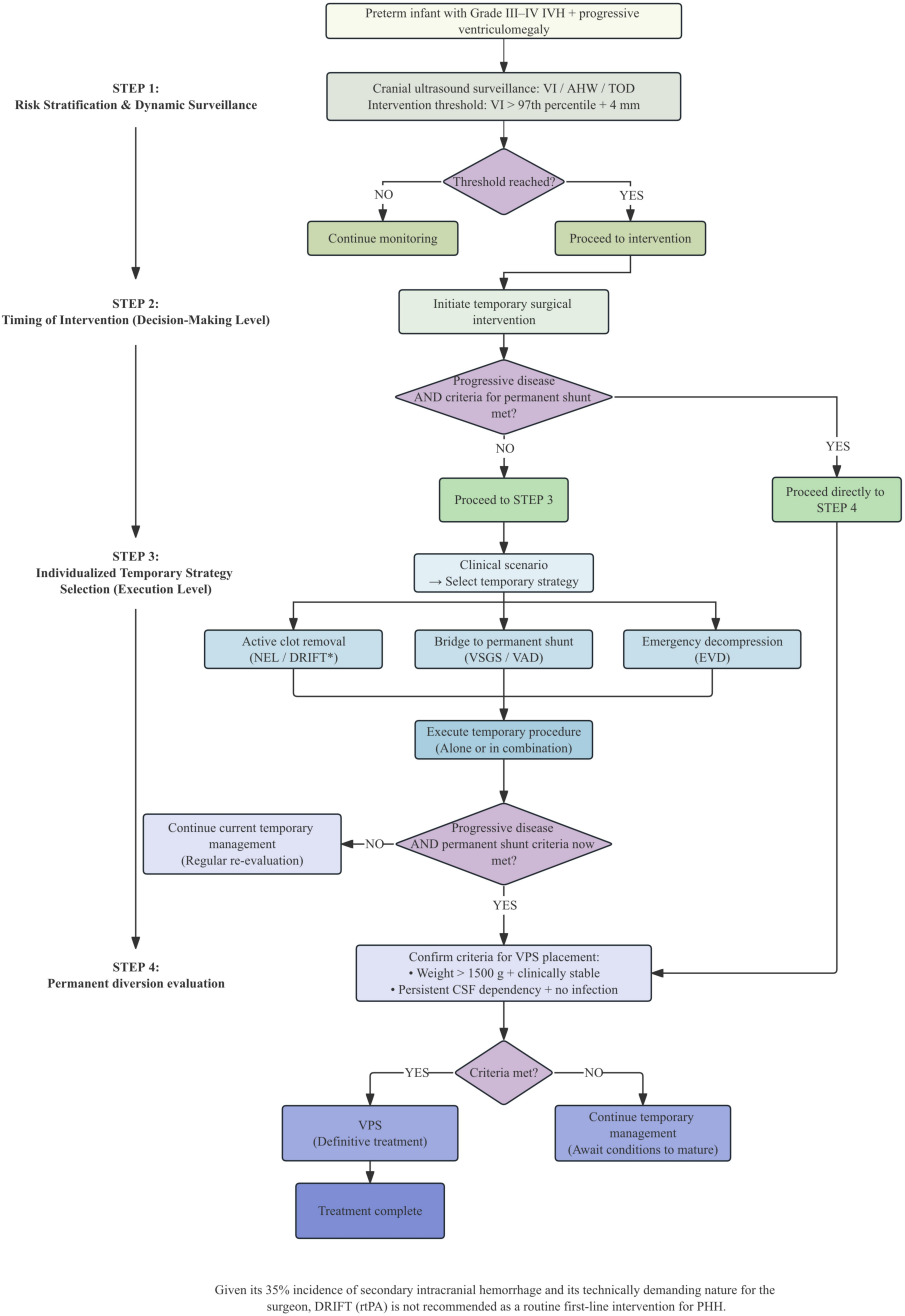
Clinical decision-making pathway for temporizing therapies in preterm infants With PHH. IVH, intraventricular hemorrhage; VI, ventricular index; AHW, anterior horn width; TOD, thalamo-occipital distance; VPS, ventriculoperitoneal shunt; NEL, neuroendoscopic lavage; DRIFT, drainage, irrigation, and fibrinolytic therapy; VSGS, ventriculosubgaleal shunt; VAD, ventricular access device; EVD, external ventricular drain; CSF, cerebrospinal fluid. * Given its 35% incidence of secondary intracranial hemorrhage and its technically demanding nature for the surgeon, DRIFT (rtPA) is not recommended as a routine first-line intervention for PHH.

Based on the available evidence, selection of a temporizing intervention may be guided by five major considerations. First, clinical urgency should be assessed. For infants with acute intracranial hypertension requiring emergency CSF diversion, EVD remains the preferred modality despite its relatively high infection and revision rates because it allows precise control of drainage pressure and flow in situations requiring continuous ICP monitoring. Second, infant weight and gestational age should be considered. VAD and VSGS are generally more suitable as primary interventions for very-low-birth-weight preterm infants weighing <1,500 g. VAD offers the advantages of bedside implantation and a relatively straightforward surgical procedure, whereas VSGS avoids repeated daily percutaneous CSF aspirationand may thereby reduce iatrogenic injury and infectious complications. Third, the anticipated duration of temporizing drainage should be considered. VAD may be more practical when CSF diversion is expected to be required for only several weeks. When prolonged temporization lasting several months or longer is anticipated, the closed-system design of VSGS may offer greater practical advantages and reduce the burden of long-term clinical monitoring. Fourth, institutional infrastructure and technical expertise are important. Although NEL may be more effective than VAD and VSGS in reducing shunt dependence and infection risk, it requires advanced neurosurgical expertise. Clinical decision-making should therefore incorporate disease severity, institutional technical capacity, and the availability of multidisciplinary collaboration. Fifth, long-term neurodevelopmental outcomes should be considered. DRIFT is the only intervention shown to improve long-term cognitive outcomes in infants with PHVD or PHH. However, its technical complexity and increased risk of hemorrhage have limited its widespread routine use. On the basis of this evidence, the present review proposes a standardized clinical decision-making pathway, as illustrated in Figure 1.

Future PHH management is expected to become increasingly precise and individualized. Decisions regarding intervention are unlikely to rely solely on a single ventricular ultrasonographic threshold. Instead, multimodal biomarkers may be integrated to enable comprehensive risk stratification. These may include white matter microstructural injury detected using diffusion tensor imaging (DTI), CSF inflammatory biomarker levels, and cerebral oxygenation parameters measured by near-infrared spectroscopy (NIRS). Such an integrated assessment framework may facilitate the accurate identification of high-risk infants most likely to benefit from early temporizing intervention (72). In parallel, regenerative approaches involving MSCs may provide pleiotropic reparative effects through immunomodulation, anti-inflammatory activity, and neurotrophic support, thereby offering a potential therapeutic strategy for perinatal brain injury.

### Summary

Substantial progress has been made in recent years in the prediction, assessment, and treatment of PHH. However, management strategies and treatment standards remain controversial. Temporizing interventions can relieve symptoms, delay disease progression, improve long-term outcomes, and reduce the need for VPS placement. Nevertheless, procedure-related complications, including infection, catheter obstruction, and secondary hemorrhage, remain important concerns. Standardized criteria for selecting temporizing procedures, protocols for diagnosing postoperative infections, and perioperative nursing guidelines are urgently needed. With advances in cranial ultrasonography and neuroendoscopy, therapeutic strategies have shifted from symptom relief toward early intervention and improvement of long-term outcomes. The potential benefits of early intervention for long-term neurological function in affected infants are now increasingly recognized. Future research should further elucidate the underlying etiology of PHH and evaluate the effectiveness of temporizing interventions, used either alone or in combination, in reducing the need for VPS placement through multicenter studies. Such efforts may facilitate the development of safer, more feasible, and more effective treatment strategies.
